# An Optimized Method to Generate Human Active Osteoclasts From Peripheral Blood Monocytes

**DOI:** 10.3389/fimmu.2018.00632

**Published:** 2018-04-04

**Authors:** Dina Abdallah, Marie-Laure Jourdain, Julien Braux, Christine Guillaume, Sophie C. Gangloff, Jacky Jacquot, Frédéric Velard

**Affiliations:** EA 4691 “Biomatériaux et Inflammation en site osseux” SFR CAP-Santé (FED 4231), Université Reims Champagne-Ardenne, Reims, France

**Keywords:** human peripheral blood mononuclear cells, osteoclasts, vitamin D3, dentin matrix resorption, CD14+ monocytes

## Abstract

Osteoclasts (OCs), the bone-resorbing cells, play a key role in skeletal development and adult bone remodeling. They also participate in the pathogenesis of various bone disorders. One of the major technical difficulties in the generation of OCs, when working on human material, is the ability to achieve large differentiation of mature OCs from human peripheral blood mononuclear cells (PBMCs). Access to a standardized source of active OCs is needed to better analyze the roles of human OCs. The aim of this study was to develop a procedure yielding active and mature OCs from fresh human PBMCs. We therefore examined the differentiation of PBMCs to OCs in different cell culture media, using non-stripped and charcoal-stripped sera in the presence of macrophage colony-stimulating factor (M-CSF) and receptor activator of nuclear factor kappa-B ligand (RANKL). We also studied the effects of vitamin D3 in the differentiation level of PBMCs to OCs. Phalloidin-AlexaFluor^®^488/DAPI fluorescent stainings and dentin resorption analyses by scanning electron microscopy were used to identify the number and size of differentiated OCs, number of nuclei per cell and resorption activities of OCs for a 7–14–21-day culture period. This study reports an optimized method for an efficient production of human active OCs from a low seeding density of PBMCs, after a 14-day culture period by using a medium containing fetal bovine charcoal-stripped serum in the presence of M-CSF and RANKL, and in the absence of vitamin D3.

## Introduction

Peripheral blood mononuclear cells (PBMCs) constitute 3–9% of the human leukocytes in the blood and thus can be easily collected and isolated in large amounts from individual human subjects. Abnormalities in PBMCs have been linked to a variety of human disorders such as hypertension (1), alcoholism (2), autoimmune liver disease (3), rheumatoid arthritis (4), and osteoporosis (5). Thus, PBMCs may represent a highly valuable cell source for functional, genomic, and epigenomic studies for dissecting the etiology mechanisms underlying various human disorders.

Peripheral blood mononuclear cells are an important source of precursors of osteoclasts (OCs), the bone-resorbing multinuclear cells, and the cytokines produced by PBMCs have profound effects on OCs differentiation, activation, and apoptosis (6). Osteoclasts are multinucleated giant cells of hematopoietic origin having a unique capacity to resorb the bone tissue (7). OCs play key roles in the regulation of bone mass and quality; a deficiency of OCs results in an increased but fragile bone state known as osteopetrosis, while excessive osteoclastic activity underlies the decreased bone mass and fragility fractures that are hallmarks of osteoporosis. Osteoporosis affects one third of women and one of eight men over the age of 50 (8), and is highly prevalent in patients with chronic inflammatory pathologies such as chronic obstructive pulmonary disease and cystic fibrosis (9–11). Unraveling the molecular program that drives the differentiation and function of OCs during the lifelong continuous cycle of bone remodeling is important for a better understanding of the pathogenesis of metabolic bone disease as well as the intricate mechanisms controlling the volume and strength of the skeleton that enables the fundamental activities of daily life (12, 13).

Reports have depicted that OCs are differentiated from PBMCs under tight regulation of osteoblasts (the forming-bone cells) (13, 14). Macrophage colony-stimulating factor (M-CSF), also known as CSF-1, is a monocyte/macrophage lineage-specific growth factor. It plays a key role in the proliferation, differentiation, and survival of OCs precursors (15). Treating PBMCs with M-CSF and receptor activator of nuclear factor kappa-B ligand (RANKL) is sufficient to induce *in vitro* OCs differentiation (16–18). More recently, an increasing number of studies reveals that OCs themselves secrete soluble factors such as sphingosine-1-phosphate (S1P), platelet-derived growth factor (PDGF)-BB, and leukemia inhibitory factor (LIF) capable to promote the differentiation of osteoblasts and bone formation (19–22). In coculture studies of osteoblasts and hematopoietic cells, active metabolites of vitamin D such as calcitriol [1,25(OH)_2_ D3] has been shown to stimulate osteoclastogenesis (23). This stimulation has been demonstrated to be due to an increase in RANKL production and consequently OCs stimulation. Hence, 1,25(OH)_2_ D3 has been believed to directly stimulate OCs resorption. As 1,25(OH)_2_ D3, and its clinically used analog, eldecalcitol, have been used therapeutically, the increase in bone mass in osteoporotic patients has been assumed to be linked to the suppression of bone resorption. Thus, the effects of 1,25(OH)_2_ D3 on osteoclastic bone resorption in *in vitro* studies seem to be opposite to *in vivo* studies. In *in vitro* studies, 1,25(OH)_2_ D3 has been shown to have both stimulatory as well as inhibitory effects on OCs activity (24, 25). These findings raised an intractable paradox regarding the action on bone of vitamin D (26). A study exploring the effects of 1,25(OH)_2_ D3 on circulating monocytes was recently published, demonstrating a reduced S1P receptor 2 expression on vitamin D-treated monocytes associated with reduced bone resorption in mice (27). Another recent study showed that treatments of OCs derived from CD14^+^ cells with active vitamin D metabolites reduces OCs size and number when cotreated with RANKL and M-CSF (28). Furthermore, data have provided new findings demonstrating that OCs are not only bone-resorbing cells but they are also involved in broader functions. OCs regulate the bone marrow niches for hematopoietic stem cells (29), B-cell progenitors, and the proliferation of malignant plasma cells (30). They are capable of driving immune T-cell response toward immunosuppression or inflammation according to their origin and to their environment (31, 32), and participate in the modulation of bone microenvironment and immune suppression in multiple myeloma (33, 34).

This short summary of the literature evidences that genomic and molecular studies using PBMCs or monocytes as a working cell models may provide novel insights into the physiopathology mechanisms underlying various immune and skeletal disorders. However, most of these studies on OCs differentiation and activity have been realized with animal models (35–37), and reports have shown opposite results in the response to cytokines between murine and human blood monocytes (38, 39). Access to a standardized source of mature human OCs from PBMCs is needed to better analyze their roles in both normal and abnormal bone regeneration and repair. Much remains to be learned before the role of human OCs in all aspects of skeletal biology can be fully appreciated. Key future directions include defining: (i) whether OCs communicate with bone-resident osteocytes to regulate remodeling, (ii) the spatiotemporal relationship of OCs-derived bone anabolic signals (i.e., bone matrix factors, clastokines and cell surface molecules) with osteoblasts and their precursors in the basic multicellular unit paradigm, and (iii) whether subsets of OCs differentially interact with other cells depending on the microenvironmental context, as suggested by recent reports (40, 41). In addition, it will be important to resolve conflicting reports, such as those that support or oppose a direct regulatory role for OCs in the HSC niche, and to add emerging data on how OCs lineage cells influence immune responses occurring at the bone interface in humans. One of the technical difficulties in the generation of OCs when working on human material, is the ability to achieve large differentiation of active and mature OCs from PBMCs. Therefore, we tested several procedures to obtain human mature OCs from PBMCs by examining the proliferation and differentiation levels of blood monocytes of healthy donors in two different cell culture media, i.e., using fetal bovine charcoal-stripped and non-stripped sera supplemented with two recombinant osteoclastogenic cytokines, M-CSF and RANKL, for a 7–14–21-day culture period. We also investigated the influence of vitamin D3 in the levels of differentiation of monocytes to active and mature OCs. This study reports an optimized method for an efficient production of human active OCs from a low seeding density of PBMCs, only after a 14-day culture period by using a medium containing fetal bovine charcoal-stripped serum (FBCSS) in the presence of M-CSF and RANKL, and in the absence of vitamin D3.

## Materials and Methods

### Reagents and Chemicals

RPMI Medium 1640 Glutamax™, Penicillin/Streptomycin 10,000 U/mL (P/S), phosphate buffer saline without Ca^2+^/Mg^2+^ (PBS), PhalloidinAlexaFluor488^®^ (Invitrogen), 4’,6-diamidino-2-phénylindole (DAPI), and ArC Amine Reactive Compensation Bead Kit were purchased from Life Technologies (Courtaboeuf, France). fetal bovine serum charcoal stripped, 1α, 25-dihydroxyvitamin D3, paraformaldehyde (PFA), bovine serum albumin (BSA), Triton X 100, and ethylenediaminetetraacetic acid (EDTA) were from (Sigma Aldrich, Saint-Quentin Fallavier, France). Polymorphprep™ was bought at Proteogenix (Schiltigheim, France). Recombinant human RANK-L and M-CSF were from Merck Millipore (Molsheim, France). Glass cover slips (Deckglaser, 12 mm of diameter) and filtrated bovine serum (FBS) (PAN Biotech™) were purchased from Dominique Dutscher (Brumath, France). RosetteSep™ Human Monocyte Enrichment Cocktail and Accutase™ were from Stem Cell Technologies (Grenoble, France). Dentine matrix comes from ImmunoDiagnostic Systems (Pouilly en auxois, France). Fluorescent mounting medium was purchased from Dako (Les Ullis, France). Mouse anti-CD66-PerCP-Cy5.5 (Clone G10F5), mouse anti-CD14-APC (clone M52E) and mouse anti-CD3-FITC (clone HIT3a), Fixable Viability Staining 510, Human BD FcBlock™, Anti-Rat and Anti-Hamster Igk/Negative Control Compensation Particles Set (BD CompBeads), Anti-Mouse Igk/Negative Control Compensation Particles Set (BD CompBeads), and plastics (tubes and plates) were from BD Biosciences (Le Pont de Claix, France).

### Isolation and Analysis of Human PBMCs and CD14^+^ Monocyte

Venous whole blood (22 mL) was drawn from each healthy volunteer of the French “Établissement Français du Sang Grand Est” (Authorization ALC/PIL/DIR/AJR/FO/606 Reims, France) by venipuncture and stored in EDTA tubes (K2E, Becton Dickinson, Le Pont de Claix, France) for further processing (*n* = 19, 15 males, 4 females, 53.8 ± 2.4 and 44.5 ± 8.2 years old, respectively). PBMCs were purified by using Polymorphprep™ density gradients: a volume of whole blood was gently added on equal volume of Polymorphprep™ in a Falcon 50-mL tube and centrifuged (1,200× *g*, 22°C, 40 min, brake off). The cell layer of PBMCs was collected in a clean Falcon 50-mL tube using a 10-mL pipette, resuspended in PBS (without Ca^2+^ and Mg^2+^) to fill the tube and centrifuged (600× *g*, 10 min, 22°C, brake on). Cell pellet was submitted to red-cell lysis by hypotonic shock. Five milliliters of cold 0.2-µm-filtered steam sterilized distilled water were used to resuspend the cells by five up and downpipetting. After 30 s, 45 mL of PBS (without Ca^2+^ and Mg^2+^) were added and cells were centrifuged (600× *g*, 10 min, 22°C, brake on). Supernatant was discarded and cell pellet was resuspended in RPMI medium 1640 Glutamax™. Cells were numbered with Malassez slide and used immediately for the differentiation process.

RosetteSep™ Human Monocyte Enrichment Cocktail was used to purify human monocytes through negative selection from whole blood. Fifty microliters of cocktail were added to each 1 mL of sample, then mixed gently by inverting the tube and then incubated for 20 min at room temperature. Cells were separated using Polymorphprep™. A volume of RosetteSep™-treated blood was gently added on an equal volume of Polymorphprep™ in a Falcon 50-mL tube and centrifuged (1,200× *g*, 22°C, 20 min, and brake off). The monocytes cell layer was collected in a clean Falcon 50-mL tube using a 10-mL pipette, resuspended in PBS (without Ca^2+^ and Mg^2+^) containing 2% of heat-inactivated FBS (v/v) to fill the tube and centrifuged (600× *g*, 10 min, 22°C, brake on). Cell pellet was resuspended in 50 mL of PBS (without Ca^2+^ and Mg^2+^) containing 2% of heat-inactivated FBS and cells were centrifuged again (600× *g*, 10 min, 22°C, brake on). Supernatant was discarded and cell pellet was resuspended in RPMI medium 1640 Glutamax™. Cells were numbered with Malassez slide and used immediately for the differentiation.

Flow cytometry was used to identify human peripheral blood populations after gradient separation with both the methods mentioned above. One million PBMCs or purified monocytes were centrifuged (600× *g*, 10 min, brake on). Supernatant was discarding by inverting the tube then pellet was stained in the remaining drop (about 50 µL) with 1-µL FVS 510 for 15 min at room temperature protected from light. Cells were washed with PBS (without Ca^2+^ and Mg^2+^) containing 2% heat-inactivated SVF and 1-mM EDTA and centrifuged at 600× *g* for 7 min, two times. Fifty microliters of specific conjugated antibodies cocktail (5 µL of anti-CD14-APC, 5 µL of CD3-FITC, 2.5 µL of anti-CD66-PerCP-Cy5.5 and 37.5 µL of PBS containing 2% heat-inactivated SVF and 1-mM EDTA) was added on the cells and incubated for 20 min at 4°C protected from light, then washed two times as described before. Cells, protected from light, were fixed with 500 µL of PFA 2% for 10 min and then washed three times. Cells were analyzed by LSR Fortessa flow cytometer working with FACSDiva software (v6.0, Becton Dickinson, Le-Pont-de-Claix, France). Side Scatter Height vs. Area plot as well as Forward Scatter Height vs. Area plot allowed the discrimination of singlet events. This population was analyzed for FVS 510 signal and high events were excluded as they correspond to died or dying cells. Lymphocytes (CD3^+^), monocytes (CD14^+^), and granulocytes (CD66^+^) subsets were identified based on the live singlets gate.

### OC Formation Assay

Cells were seeded on glass cover slips (12 mm of diameter) into 24-well plates. A density of 5 × 10^5^ cells per well was used for monocytes. A low seeding density of 1.5 × 10^6^ cells per well was used for PBMCs to take into account the presence of lymphocytes in the cell count. The latter are non-adherent cells which were removed by medium renewal. Both the cell populations were cultured in RPMI medium 1640 Glutamax™, supplemented with 10% of FBS charcoal-stripped or 10% of FBS (v/v), 25 ng.mL^−1^ of recombinant human M-CSF and 100 ng mL^−1^ of recombinant human RANK-L with or without 10 nM of 1α, 25-dihydroxyvitamin D3, for 7–14 and 21 days at 37°C, in humidified atmosphere containing 5% CO_2_ over time. The medium was removed and replaced with fresh medium each 48–72 h. At the end of 7-, 14-, and 21-day periods, supernatants were removed and cells were fixed with 4% of PFA (v/v in PBS without Ca^2+^ and Mg^2+^) for 10 min at 37°C, then permeabilized with Triton X100 0.5% (v/v in PBS without Ca^2+^ and Mg^2+^) for 15 min at room temperature. After two washes of 5 min in PBS (without Ca^2+^ and Mg^2+^), actin cytoskeleton was stained with AlexaFluor^®^488-conjugated Phalloidin (1/100 v:v) diluted into BSA 0.5% (v:v in PBS without Ca^2+^ and Mg^2+^) for 30 min at room temperature protected from light. Cells were then washed with PBS without Ca^2+^ and Mg^2+^ two times for 5 min. The nuclei were labeled with DAPI 1/30,000 (v: v in distilled water) for 5 min at room temperature protected from light, then washed two times with distilled water. Cells were visualized by fluorescent microscopy. Each analysis was performed on five randomly chosen microscope fields (×20 magnification, Zeiss Axiovert 200M, Axiovision Software, Zeiss, Marly-le-Roi, France). Images were processed with ImageJ software (v1.50i, NIH, USA). For the counting of OC cells and their nuclei, cell counter plugin was used. Actin ring positive cells bearing three or more nuclei were considered to be OCs. For the area measurement, we used freehand selection tool. For nuclei count and area measurements, at least 21 cells were analyzed per condition.

### OC Resorption Assay

In order to evidence OC resorption activity, we transferred the cells after 14 days of differentiation onto dentin matrix. The latter was previously incubated in serum-free RPMI medium 1640 Glutamax™ 1 h at room temperature for rehydration purpose. Culture supernatants were discarded and cells were rinsed with PBS (without Ca^2+^ and Mg^2+^) before being detached using Accutase™ for 30 min at 37°C. Detached cells were collected and rinsed in PBS (without Ca^2+^ and Mg^2+^), centrifuged (600× *g*, 10 min, brake on), and then counted. Fifty thousand cells were deposited on dentin matrix for a week in differentiation medium as described above. After culture time, dentin matrix was washed with PBS two times and incubated with NaOH (1M) at room temperature for 20 min with 30-s vortex every 5 min. Matrix dentin was dried and kept at 37°C. Before SEM observation, samples were sputtered with thin gold–palladium film under a JEOL ion sputter JFC 1100. Cells were viewed using a LaB6 electron microscope (JSM-5400 LV, JEOL, Croissy, France).

### Statistical Analyses

The significance of the biological results was assessed with a non-parametric approach owing to a lack of normal distribution of the assessed variables. Exact non-parametric and stratified (when appropriate) Wilcoxon Mann–Whitney tests were used (StatXact 7.0, Cytel Inc., Cambridge, MD, USA). A value of *p* < 0.05 was accepted as statistically significant. In all figures, red bar represents the median value, limits of the boxes represent the first and third quartile, and bars represent the first and ninth decile. At least four different donors were used for each experiment.

## Results and Discussion

### Use of FBCSS Early Generates Large Numbers of Human OCs From PBMCs

**Figure 1:**
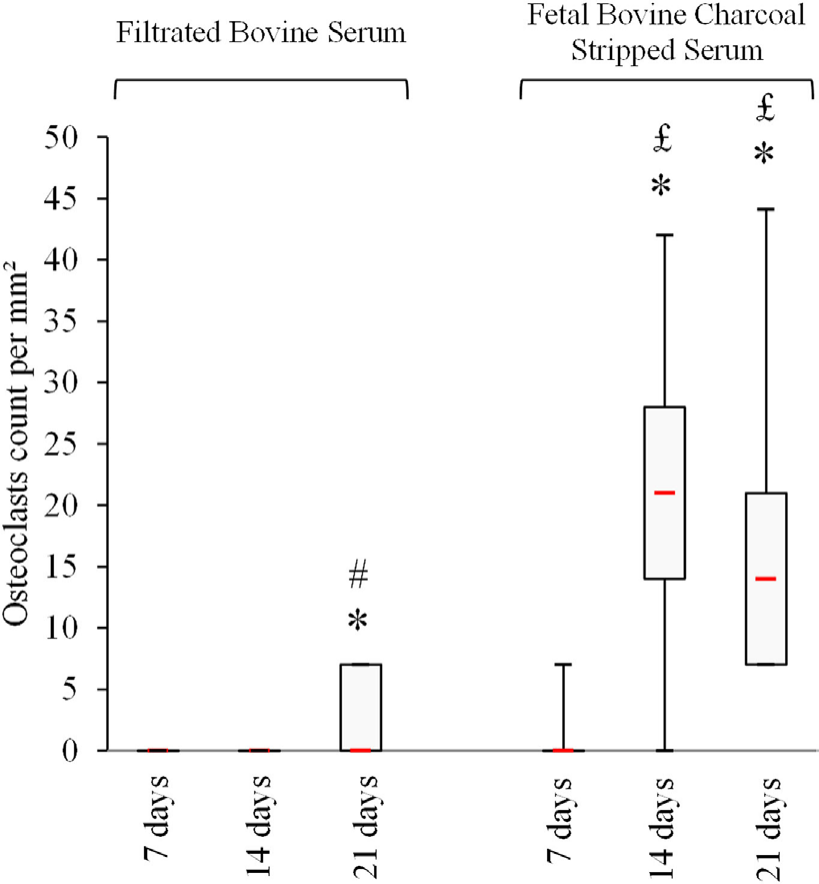
Fetal bovine charcoal-stripped serum allowing earlier and greater osteoclasts formation *in vitro*. Osteoclasts count at each time point is shown.* *p* < 0.05 vs. 7 days, ^#^ *p* < 0.05 vs. 14 days, and ^£^ *p* < 0.05 vs. filtrated bovine serum.

Peripheral blood mononuclear cells from fresh whole blood of normal donors were isolated with the Polymorphprep™ protocol according to the manufacturer’s guidelines. To obtain a large differentiation of mature human OCs from PBMCs *in vitro* culture, we initially tested two bovine sera, i.e., FBS and FBCSS in the presence of M-CSF (25 ng/mL) and RANK-L (100 ng/mL) for a 7–14–21-day culture period. The idea was to evaluate the effects of the absence of lipids (with the use of FBCSS) in the proliferation and differentiation of PBMCs to OCs. As shown in Figure 1, the culture with FBCSS promptly generated large numbers of OCs (23.8 ± 10.4 OCs per mm^2^) for the 14-day time point compared with the FBS (0.35 ± 0.78 OCs per mm^2^) at the same period. At the 21-day time point, the number of OCs was always greater with the FBCSS (20.3 ± 7.9 OCs per mm^2^) compared with the FBS (3.88 ± 4.19 OCs per mm^2^). Thus, there is an important improvement of the generation of OCs using FBCSS, as opposed to FBS. We therefore used the FBCSS in all the following experiments.

### Use of Purified CD14^+^ Monocytes Does Not Improve the Yield of Differentiated OCs

**Figure 2:**
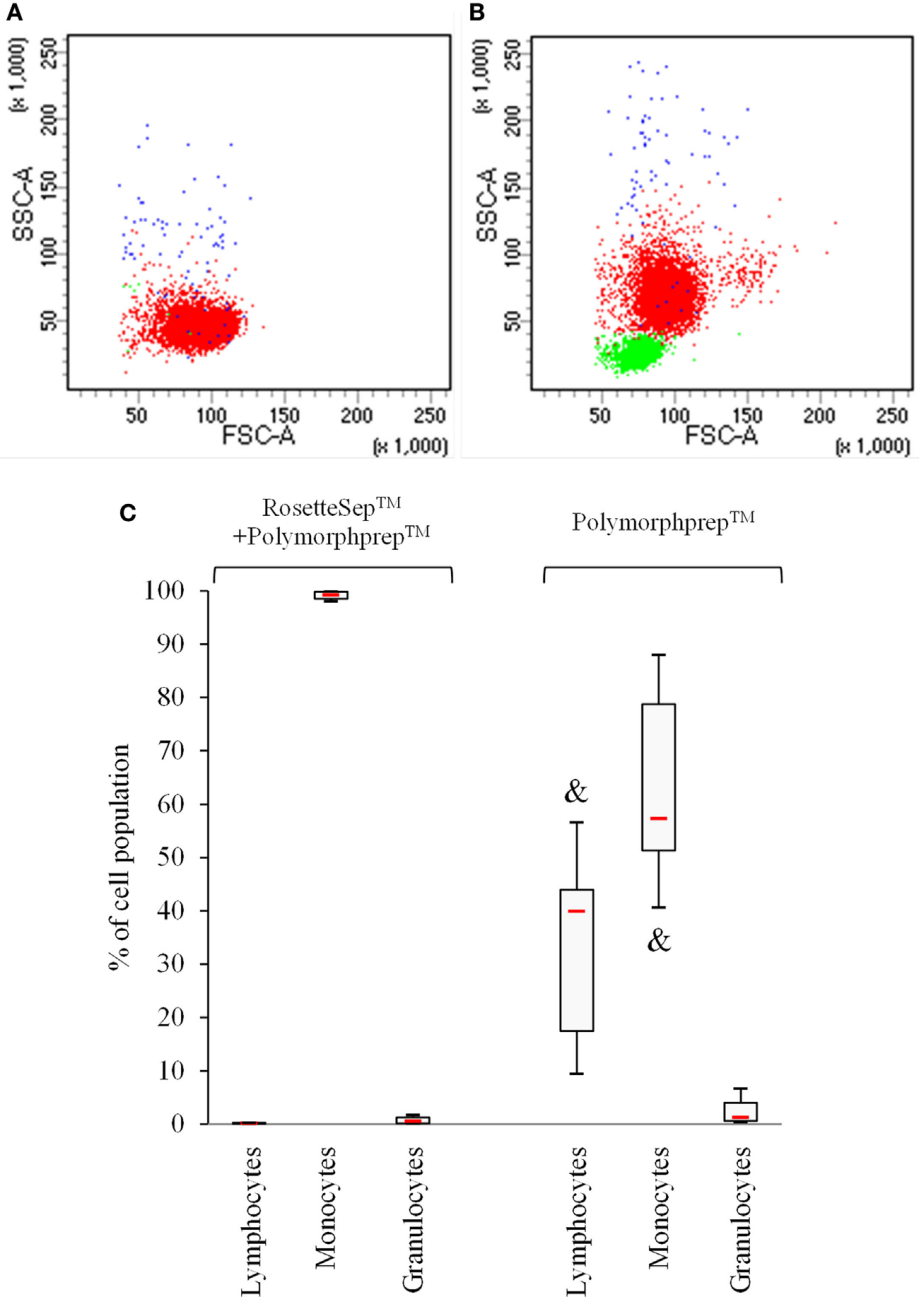
Use of RosetteSep™ on total blood samples prior to Polymophprep™ protocol allows to obtain a higher monocytic population purity. Peripheral blood mononuclear cells were analyzed by flow cytometry. CD3^+^ (lymphocytes, green dots), CD14^+^ (monocytes, red dots), and CD66^+^ (granulocytes, blue dots) cell contents were monitored. Representative dot plots **(A,B)** and cell population percentage **(C)** are shown. ^&^ *p* < 0.05 vs. RosetteSep™ + Polymorphprep™.

In order to gain the greater number of mature OCs, we investigated the potential effects of the use of CD14^+^ monocytes-enriched cell population compared with the use of PBMCs for a 7–14–21-day culture period. The CD14^+^ monocytes were purified from fresh whole blood by negative selection with RosetteSep™ human monocyte-enrichment mixture, before performing the Polymorphprep™ protocol for the separation of cell populations. As expected, we observed a 98% purity of CD14^+^ monocytes evaluated by flow cytometry after the use of both RosetteSep™ and Polymorphprep™ protocols (Figures 2A,C) compared with a 58% purity of CD14^+^ monocytes (40% lymphocytes and 2% granulocytes) with the use of Polymorphprep™ protocol alone (Figures 2B,C).

**Figure 3:**
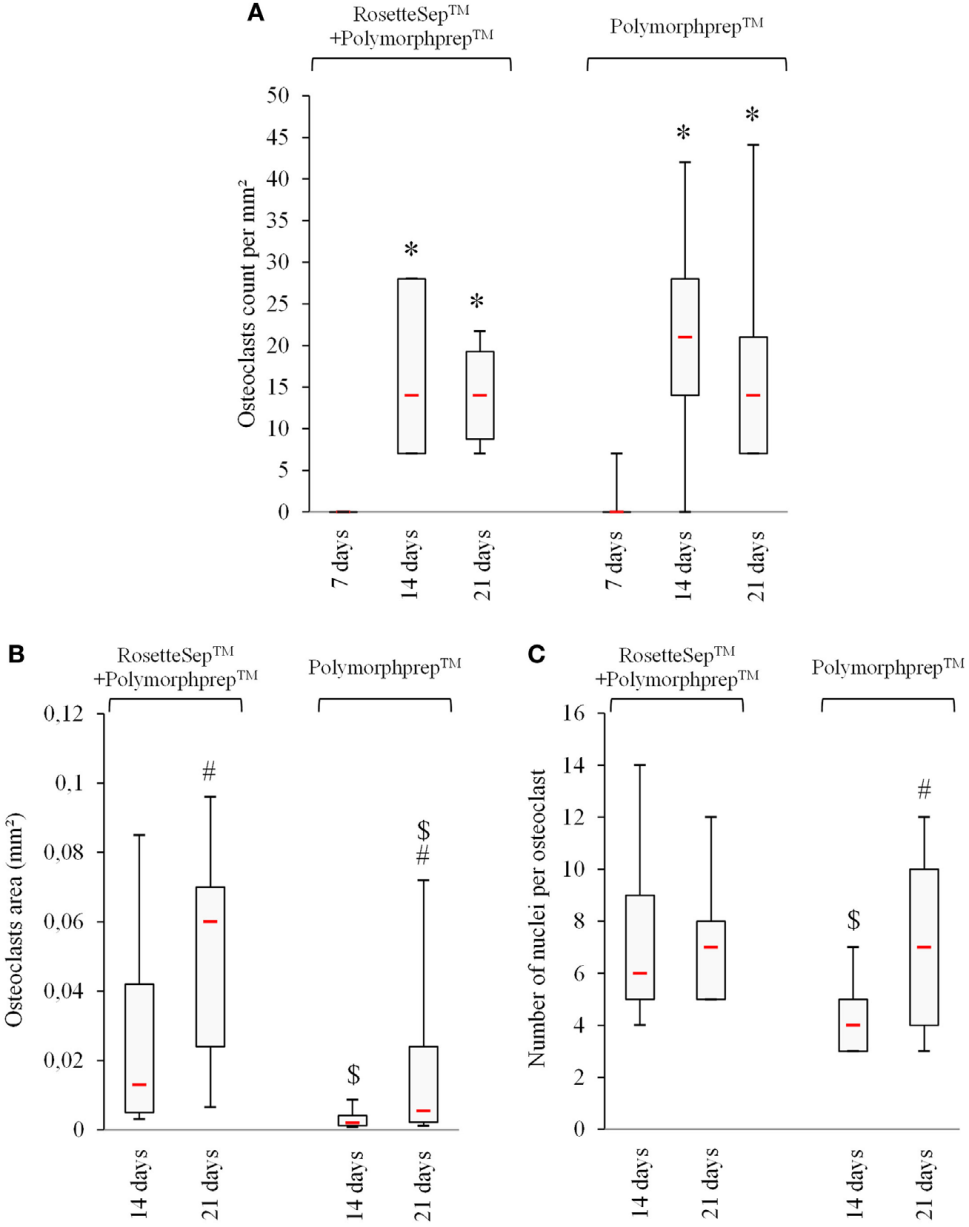
The use of monocytes-enriched cell population does not ameliore osteoclastogenesis *in vitro*. **(A)** Number of actin ring positive and multinucleated cells, **(B)** evaluation of osteoclasts area, and **(C)** number of nuclei per osteoclast are shown. * *p* < 0.05 vs. 7 days, ^#^ *p* < 0.05 vs. 14 days, and ^$^ *p* < 0.05 vs. RosetteSep™ + Polymorphprep™.

We next plated the two cell populations (obtained either after combined RosetteSep™ and Polymorphprep™ protocols, or after the Polymorphprep™ protocol alone) in the FBCSS supplemented with M-CSF (25 ng/mL) and RANK-L (100 ng/mL) for a 7–14–21-day culture period. To characterize the osteoclastogenesis parameters from these two cell populations, we evaluated the number of generated OCs, the size of OCs and the number of nuclei/OC on the 7–14–21-day time points (Figures 3A–C). There was no noteworthy difference in the number of OCs. The size of OCs was reduced in Polymorphprep™ protocol alone-derived OCs both at 14 and 21 days and the number of nuclei per OC was also minimized in this condition but only after 14 days. No significant difference could be observed after 21 days. Thus, the pre-purification of CD14^+^ monocytes by the RosetteSep™ step prior to the Polymorphprep™ protocol showed no advantageous change in fold expansion of generated OCs. We suggest that other cell types as lymphocytes present in PBMCs population could act as early enhancers for osteoclastogenesis, such as T cells which are well known to secrete RANKL. In light of our results, Polymorphprep™ protocol was used for subsequent experiments.

### The Addition of 10-nM 1,25(OH)_2_ D3 Delaying the Osteoclastogenesis and Decreasing the Number of Nuclei Per OC

**Figure 4:**
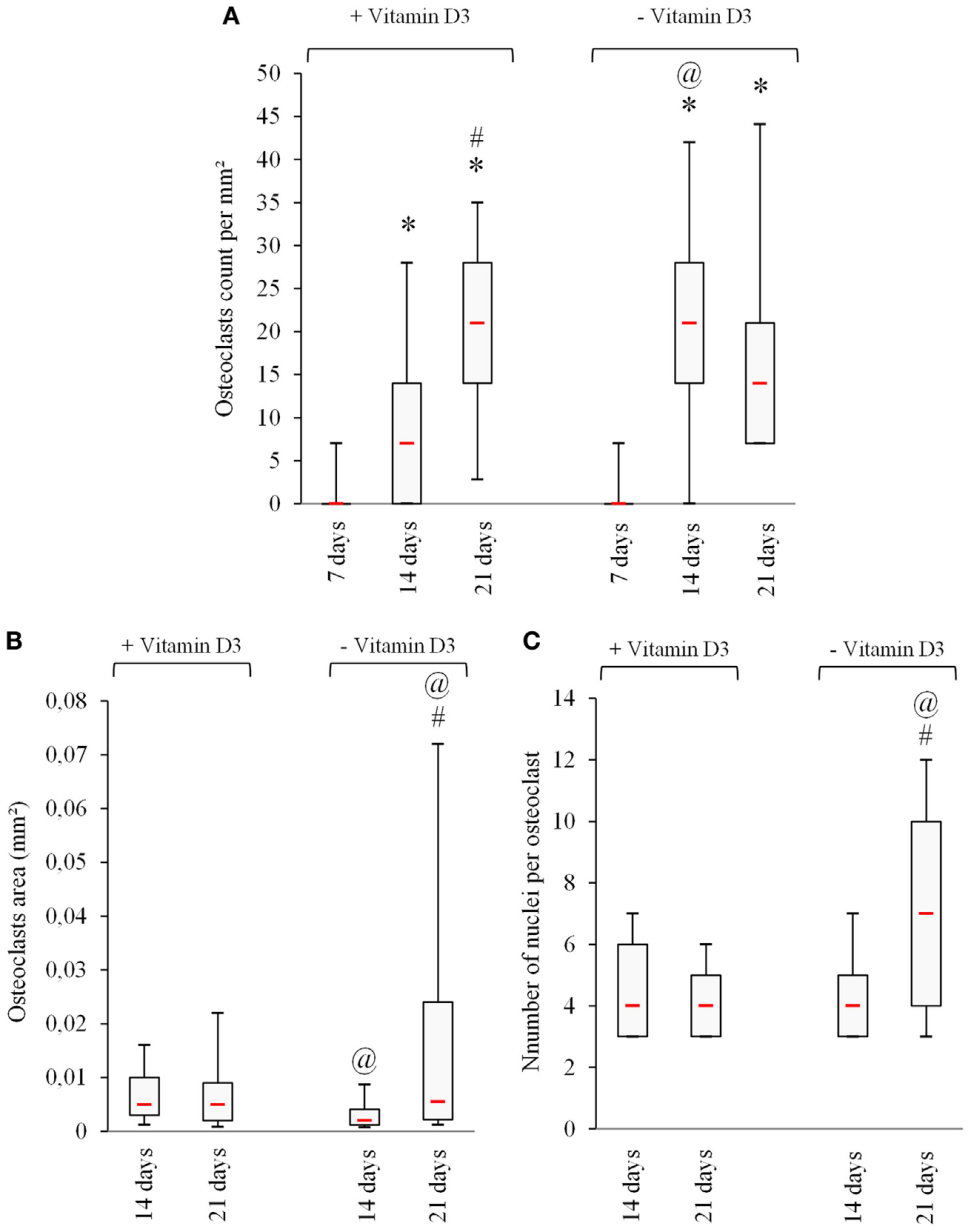
The addition of vitamin D3 delays osteoclastogenesis *in vitro*. **(A)** Number of actin ring positive, **(B)** multinucleated cells evaluation of osteoclasts area, and **(C)** number of nuclei per osteoclast are shown. * *p* < 0.05 vs. 7 days, ^#^ *p* < 0.05 vs. 14 days, and ^@^ *p* < 0.05 vs. + vitamin D3.

Seeking to improve the understanding of the effects of vitamin D3 on the proliferation and differentiation of OCs, we have studied the effects of a low concentration of 1,25(OH)_2_ D3 (10 nM) on the number of OCs, the size of OCs, and the number of nuclei per OC, generated from PBMCs in the FBCSS supplemented with M-CSF (25 ng/mL) and RANK-L (100 ng/mL) for a 7–14–21-day culture period. As demonstrated in Figure 4A, the presence of 10-nM 1,25(OH)_2_ D3 delayed the OCs generation within a 14-day culture period (average 9.2 ± 3.8 OCs/mm^2^) compared with the number of OCs generated in the absence of 1,25(OH)_2_ D322 (average 23.8 ± 10.4 OCs /mm^2^), but this was not observed after a 21-day culture (average 19.4 ± 4.7 OC s/mm^2^ vs. 20.3 ± 7.9 OC s/mm^2^), respectively. The OC size and number of nuclei per OC that is an indication of how many OC precursors may have fused together was examined in response to 10-nM vitamin D3. No significant change was observed in the OC size according the presence or the absence of vitamin D3, except a tendency toward a lower size at 14 days without 1,25(OH)_2_ D3 which was corrected after 21 days. A significantly greater size of OCs with increased number of nuclei was demonstrated in the absence of vitamin D3 at the 21-day time point (Figures 4B,C).

Altogether, we find that the addition of 10-nM 1,25(OH)_2_ D3 delayed osteoclastogenesis and reduced the OCs size and number of nuclei per OC. Our findings are in good agreement with recent reports on 1,25(OH)_2_ D3 showing that lower concentrations [0.1 and 0.5-nM 1,25(OH)_2_ D3] dose-dependently decreased the number of human OCs (28).

### Resorptive Activity of OCs Generated Only After a 14-Day Period

**Figure 5:**
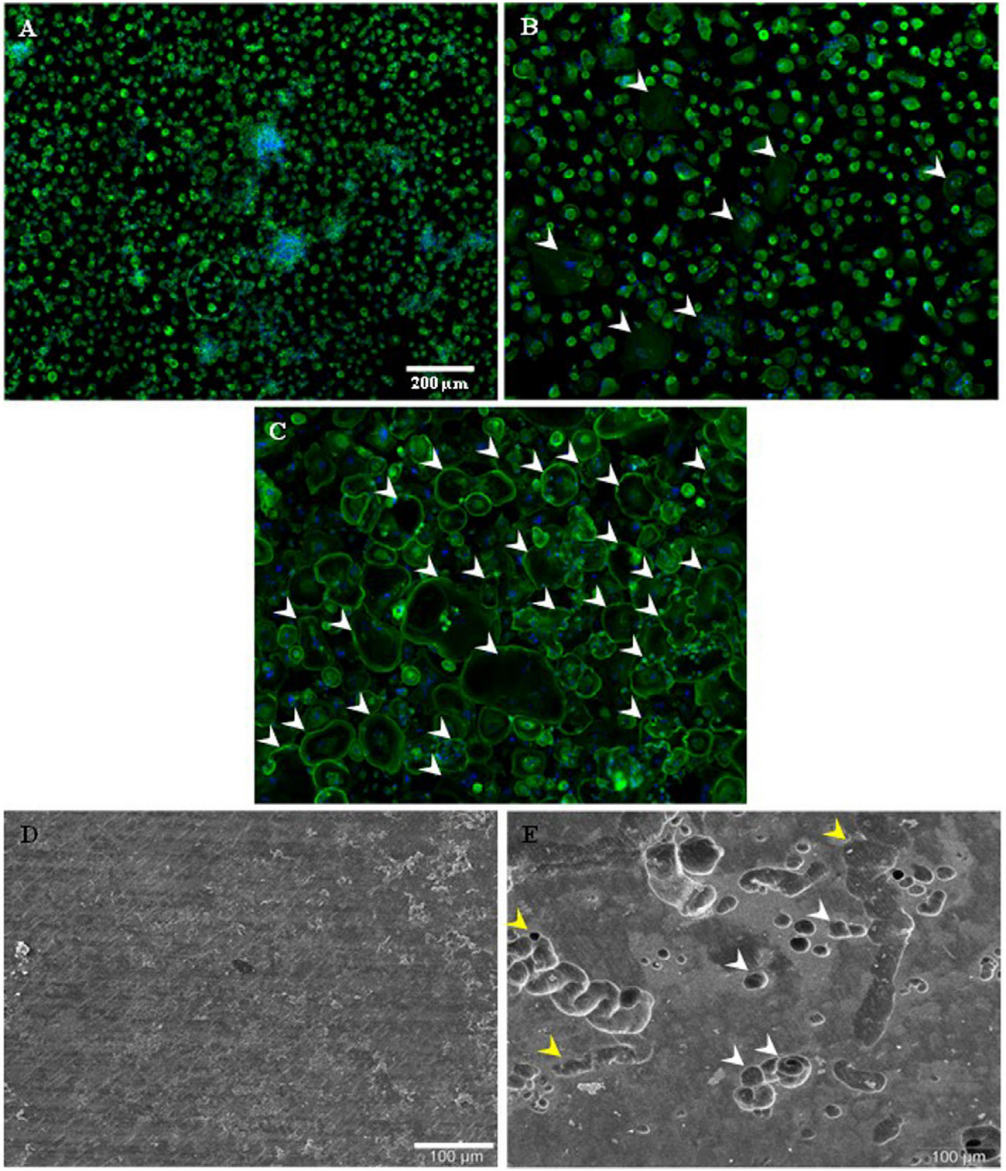
Kinetic of monocytic-precursors fusion toward osteoclastic phenotype and osteoclast-resorbing activity on dentin disks. Phalloidin-AlexaFluor^®^488/DAPI stainings on freshly isolated human primary peripheral blood mononuclear cells (Polymorphprep™ protocol) are shown for 7 **(A)**, 14 **(B)**, and 21 **(C)** days culture periods. Some osteoclasts are highlighted with white arrowheads. Scale bar = 200 µm. Cells plated on dentin disk for 7 **(D)** and 14 **(E)** were observed by SEM. Pits (white arrowheads) and trenches (yellow arrowheads) are visible. Scale bar = 100 µm.

**Figure 6:**
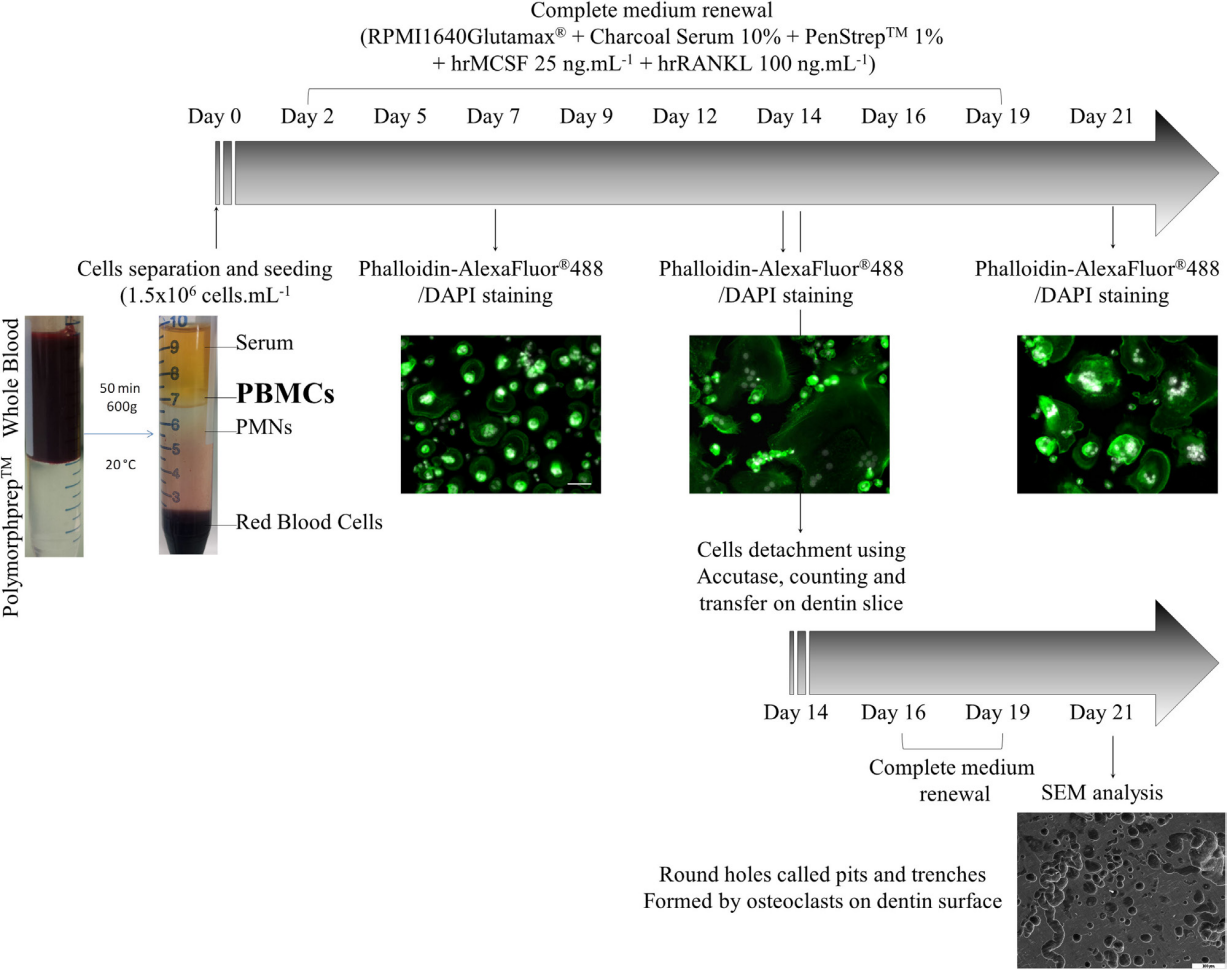
A flow chart of our procedure to generate human osteoclasts from peripheral blood mononuclear cells (PBMCs).

To determine the functional activity of OCs generated from PBMCs, their resorptive activity on dentin disks was examined when cultured in FBCSS supplemented with M-CSF (25 ng/mL) and RANK-L (100 ng/mL) and without vitamin D3 for a 7–14–21-day culture period. Figures 5A,C illustrate the kinetic of OCs differentiation from PBMCs examined by light microscopy for the 7–14–21-day culture period. In our culture conditions, we observed a large number of OCs generated only after a 14-day period (Figure 5B). We therefore investigated the resorptive activities of OCs generated after 7 and 14 days of culture and after being replated on dentin disks for an additional 7-day period. By examination with scanning electron microscopy, no degradation of dentin matrix was observed when PBMCs were cultured for a 7-day period (Figure 5D). On the contrary, OCs generated from PBMCs after a 14-day period were able to resorb dentin. As shown in Figure 5E, the formation of round holes called “pits” and trenches was well observed on the dentin surface. The distinction between the trench- and pit-resorption mode was previously defined as OCs being able to resorb while migrating, thereby generating trenches which reflect long periods of resorption parallel to the bone surface. The trench mode was recently proposed to involve high rates of collagenolysis vs. demineralization by OCs, as investigated through pharmacological manipulation (42, 43). A flow chart of our procedure to generate human OCs from PBMCs is depicted in the Figure 6.

In summary, we describe an optimized method for an efficient production of human active OCs from PBMCs, bearing the main characteristic parameters of OCs and retaining their resorptive activities. The strength of our method is to generate human OCs by using a low seeding density of PBMCs (1.5 × 10^6 ^/mL) and to be an easy and inexpensive procedure, as the use of human monocyte enrichment (i.e., RosetteSep™ human monocyte-enrichment mixture) and the supplementation of vitamin D3 are not necessary. This method opens the way for investigating the basic mechanisms underlying the proliferation and differentiation of monocyte precursors/OCs from PBMCs obtained from patients with bone disorders such as osteoporosis, and in patients with chronic inflammatory pathologies such as chronic obstructive pulmonary disease and cystic fibrosis.

## Ethics Statement

This study was carried with the recommendations of the University Reims Champagne Ardenne review board with written informed consent from all subjects in accordance with the declaration of Helsinki.

## Author Contributions

DA, M-LJ, JB, CG, and SG were involved in conception and design of the study and methodology, experiments, and acquisition of data. JJ and FV participated in conception and design of the study, methodology and acquisition of data, and manuscript writing and review. All authors have read and approved the final manuscript.

## Conflict of Interest Statement

The authors declare that the research was conducted in the absence of any commercial or financial relationships that could be construed as a potential conflict of interest.
